# Bis(2-methoxy­phenolato-κ^2^ *O*,*O*′)copper(II)

**DOI:** 10.1107/S160053680903582X

**Published:** 2009-09-12

**Authors:** Guo-Zhu Mao, Xiu-Lian Nong, Shu Hua Zhang

**Affiliations:** a School of Environmental Science and Technology, Tianjin University, Tianjin 300072, People’s Republic of China; b Department of Chemistry and Chemical Engineering, Guangxi Normal University, Guilin 541004, People’s Republic of China; c School of Chemistry and Bioengineering, Guilin University of Technology, Guilin 541004, People’s Republic of China

## Abstract

In the title compound, [Cu(C_7_H_7_O_2_)_2_], the asymmetric unit contains one and a half molecules with the central Cu(II) atoms situated on a general position and on a centre of inversion, respectively. Both Cu(II) atoms show a similar slightly distorted square-planar coordination, resulting from four O atoms of two 2-methoxyphenolate anions.

## Related literature

For 2-meth­oxy-phenol compounds, see: Campello *et al.* (1997 ▶); Floriani *et al.* (1988 ▶); Minhas *et al.* (1993 ▶); Kuo *et al.* (1999 ▶); Schumann *et al.* (1996 ▶); Sobota *et al.* (2001 ▶).
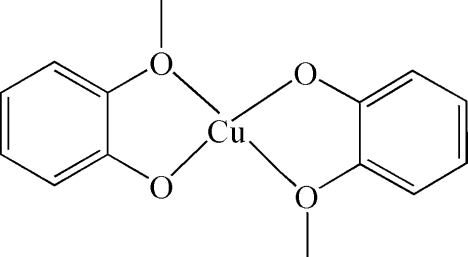


## Experimental

### 

#### Crystal data

[Cu(C_7_H_7_O_2_)_2_]
*M* *_r_* = 333.82
Triclinic, 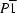

*a* = 9.5190 (19) Å
*b* = 11.540 (2) Å
*c* = 12.488 (3) Å
α = 102.83 (3)°
β = 103.93 (3)°
γ = 111.20 (3)°
*V* = 1166.7 (6) Å^3^
*Z* = 3
Mo *K*α radiation
μ = 1.42 mm^−1^
*T* = 293 K
0.23 × 0.12 × 0.08 mm

#### Data collection

Bruker P4 diffractometer
Absorption correction: multi-scan (*SADABS*; Sheldrick, 1996 ▶) *T*_min_ = 0.656, *T*_max_ = 0.857
6930 measured reflections
4164 independent reflections
3466 reflections with *I* > 2σ(*I*)
*R*_int_ = 0.037

#### Refinement

*R*[*F* ^2^ > 2σ(*F* ^2^)] = 0.051
*wR*(*F* ^2^) = 0.129
*S* = 0.93
4164 reflections
289 parameters
4 restraints
H-atom parameters constrained
Δρ_max_ = 0.70 e Å^−3^
Δρ_min_ = −0.63 e Å^−3^

### 

Data collection: *XSCANS* (Bruker, 1997 ▶); cell refinement: *XSCANS*; data reduction: *SHELXTL* (Sheldrick, 2008 ▶); program(s) used to solve structure: *SHELXS97* (Sheldrick, 2008 ▶); program(s) used to refine structure: *SHELXL97* (Sheldrick, 2008 ▶); molecular graphics: *SHELXTL*; software used to prepare material for publication: *SHELXTL*.

## Supplementary Material

Crystal structure: contains datablocks I, global. DOI: 10.1107/S160053680903582X/gw2068sup1.cif

Structure factors: contains datablocks I. DOI: 10.1107/S160053680903582X/gw2068Isup2.hkl

Additional supplementary materials:  crystallographic information; 3D view; checkCIF report

## Figures and Tables

**Fig. 1.**
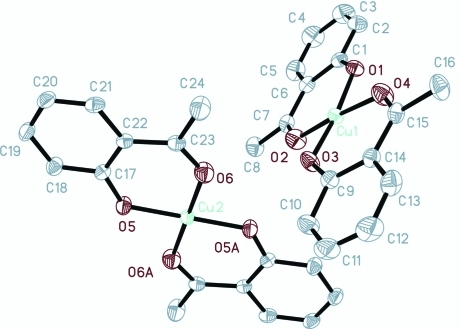
The molecular structure of (I), showing 30% probability displacement ellipsoids. Symmetry codes: (A) -x + 1, -y + 2, -z + 1.

**Fig. 2.**
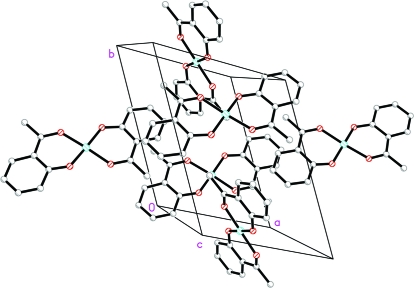
Packing diagram of title complex,hydrogen atoms were omitted.

## supplementary crystallographic information

### Comment

2-Methoxy-phenol ligand can act as either mondentate ligand (Campello, *et al.*, 1997), or didentate ligand (Sobota, *et al.*, 2001), or mu_2_-o ligand or mu_3_:eta^1^:eta^2^-O ligand (Schumann, *et al.* 1996) or mu_4_:eta^1^:eta^3^-O ligand (Floriani, *et al.* 1988). However, copper compound with 2-Methoxy-phenol have not been reported till today (http://www.ccdc.cam.ac.uk/). The title compound, (I), is a new Cu^II^ complex prepared by reaction of 2-Methoxy-phenol and Copper(II) nitrate using solvothermal technique.

There are one CuÎI^ atom and two *L*^-^ ligand in the asymetric unit. The Cu^II^ atom has a slightly distorted square-planar environment, formed by four O atoms from two different *L*^-^ ligands. The *L*^-^ ligand binds to copper in a didentate mode, through two O atoms. In the title complex, the two copper lied in the different position that the Cu2 is at the center of symmetry (010) plane and the Cu1 is at a general position (Fig. 2). The complex further constructed a 3-D network through very weak C–H···O hydrogen bond (C21–H21···O1^i^, 3.426 (1) Å, symmetry code: (i) 1 - *y*,2 - *y*,1 - *z*) and C–H···p hydrogen bond (C16···P^ii^, 3.652 (1) Å, symmetry code: (ii) 1 + *x*, *y*, *z*).

### Experimental

A solution of (0.124 g, 1 mmol) 2-Methoxy-phenol and (0.056 g, 1 mmol) potassium hydroxide in 8 ml absolute methanol was added ((0.125 g, 0.5 mmol) Copper nitrate tetrahydrate. The solution was placed in a 15-ml Tetlon-lined stainless steel parr bomb. The bomd was heated at 363 k for 96 h. The cooled mixture yielded blue block-shaped crystal of (1) in about 71% yield. The crystals were washed with methanol and then dried in air.

### Refinement

H atoms were positioned geometrically and refined with a riding model, with distances 0.96 Å(CH_3_) or 0.93 Å(aromatic ring). and with *U*_iso_(H) = 1.2 *U*_eq_(aromatic ring) or *U*_iso_(H) = 1.5 *U*_eq_((CH_3_).

### Crystal data

**Table:**

[Cu(C_7_H_7_O_2_)_2_]	*Z* = 3
*M**_r_* = 333.82	*F*(000) = 513
Triclinic, *P*1	*D*_x_ = 1.425 Mg m^−^^3^
Hall symbol: -P 1	Mo *K*α radiation, λ = 0.71073 Å
*a* = 9.5190 (19) Å	Cell parameters from 4216 reflections
*b* = 11.540 (2) Å	θ = 3.1–25.3°
*c* = 12.488 (3) Å	µ = 1.42 mm^−^^1^
α = 102.83 (3)°	*T* = 293 K
β = 103.93 (3)°	Block, blue
γ = 111.20 (3)°	0.23 × 0.12 × 0.08 mm
*V* = 1166.7 (6) Å^3^	

### Data collection

**Table:**

Bruker P4 diffractometer	4164 independent reflections
Radiation source: fine-focus sealed tube	3466 reflections with *I* > 2σ(*I*)
graphite	*R*_int_ = 0.037
ω scans	θ_max_ = 25.3°, θ_min_ = 3.1°
Absorption correction: multi-scan (*SADABS*; Sheldrick, 1996)	*h* = −11→11
*T*_min_ = 0.656, *T*_max_ = 0.857	*k* = −13→13
6930 measured reflections	*l* = −14→12

### Refinement

**Table:**

Refinement on *F*^2^	Primary atom site location: structure-invariant direct methods
Least-squares matrix: full	Secondary atom site location: difference Fourier map
*R*[*F*^2^ > 2σ(*F*^2^)] = 0.051	Hydrogen site location: inferred from neighbouring sites
*wR*(*F*^2^) = 0.129	H-atom parameters constrained
*S* = 0.93	*w* = 1/[σ^2^(*F*_o_^2^) + (0.0653*P*)^2^ + 1.5*P*] where *P* = (*F*_o_^2^ + 2*F*_c_^2^)/3
4164 reflections	(Δ/σ)_max_ = 0.001
289 parameters	Δρ_max_ = 0.70 e Å^−^^3^
4 restraints	Δρ_min_ = −0.63 e Å^−^^3^

### Special details

**Table:**

Geometry. All e.s.d.'s (except the e.s.d. in the dihedral angle between two l.s. planes) are estimated using the full covariance matrix. The cell e.s.d.'s are taken into account individually in the estimation of e.s.d.'s in distances, angles and torsion angles; correlations between e.s.d.'s in cell parameters are only used when they are defined by crystal symmetry. An approximate (isotropic) treatment of cell e.s.d.'s is used for estimating e.s.d.'s involving l.s. planes.
Refinement. Refinement of *F*^2^ against ALL reflections. The weighted *R*-factor *wR* and goodness of fit *S* are based on *F*^2^, conventional *R*-factors *R* are based on *F*, with *F* set to zero for negative *F*^2^. The threshold expression of *F*^2^ > σ(*F*^2^) is used only for calculating *R*-factors(gt) *etc*. and is not relevant to the choice of reflections for refinement. *R*-factors based on *F*^2^ are statistically about twice as large as those based on *F*, and *R*- factors based on ALL data will be even larger.

### Fractional atomic coordinates and isotropic or equivalent isotropic displacement parameters (Å^2^)

**Table:**

	*x*	*y*	*z*	*U*_iso_*/*U*_eq_	
Cu1	0.52201 (5)	0.70662 (4)	0.76824 (4)	0.03605 (16)	
Cu2	0.5000	1.0000	0.5000	0.03477 (19)	
C1	0.1962 (4)	0.5274 (4)	0.7391 (3)	0.0393 (9)	
C2	0.0931 (5)	0.4117 (4)	0.7551 (4)	0.0544 (11)	
H2	0.1388	0.3665	0.7934	0.065*	
C3	−0.0719 (6)	0.3655 (5)	0.7154 (5)	0.0700 (14)	
H3	−0.1355	0.2901	0.7273	0.084*	
C4	−0.1437 (6)	0.4304 (6)	0.6579 (5)	0.0746 (15)	
H4	−0.2552	0.3979	0.6298	0.090*	
C5	−0.0486 (5)	0.5435 (5)	0.6426 (4)	0.0628 (13)	
H5	−0.0982	0.5867	0.6046	0.075*	
C6	0.1233 (4)	0.5970 (4)	0.6827 (3)	0.0418 (9)	
C7	0.2162 (4)	0.7193 (4)	0.6650 (3)	0.0419 (9)	
C8	0.1304 (5)	0.7895 (5)	0.6067 (4)	0.0573 (12)	
H8A	0.2086	0.8694	0.6053	0.086*	
H8B	0.0672	0.8107	0.6502	0.086*	
H8C	0.0613	0.7328	0.5278	0.086*	
C9	0.8449 (4)	0.8867 (4)	0.7933 (4)	0.0414 (9)	
C10	0.9435 (5)	1.0056 (4)	0.7786 (4)	0.0559 (11)	
H10	0.8951	1.0450	0.7342	0.067*	
C11	1.1084 (6)	1.0620 (5)	0.8293 (5)	0.0699 (14)	
H11	1.1699	1.1386	0.8180	0.084*	
C12	1.1844 (5)	1.0075 (5)	0.8966 (5)	0.0727 (15)	
H12	1.2960	1.0477	0.9315	0.087*	
C13	1.0938 (5)	0.8930 (5)	0.9118 (4)	0.0605 (12)	
H13	1.1464	0.8563	0.9565	0.073*	
C14	0.9226 (4)	0.8284 (4)	0.8617 (3)	0.0410 (9)	
C15	0.8344 (4)	0.7091 (4)	0.8844 (3)	0.0425 (9)	
C16	0.9244 (6)	0.6458 (5)	0.9492 (5)	0.0648 (13)	
H16A	0.8485	0.5657	0.9520	0.097*	
H16B	0.9902	0.7058	1.0277	0.097*	
H16C	0.9915	0.6259	0.9089	0.097*	
C17	0.4664 (4)	0.8676 (4)	0.2612 (3)	0.0362 (8)	
C18	0.4051 (5)	0.8508 (4)	0.1408 (3)	0.0453 (9)	
H18	0.3634	0.9074	0.1197	0.054*	
C19	0.4051 (5)	0.7535 (4)	0.0537 (4)	0.0472 (10)	
H19	0.3619	0.7437	−0.0247	0.057*	
C20	0.4707 (5)	0.6694 (4)	0.0840 (4)	0.0465 (10)	
H20	0.4724	0.6042	0.0259	0.056*	
C21	0.5329 (4)	0.6839 (4)	0.2007 (4)	0.0423 (9)	
H21	0.5776	0.6283	0.2197	0.051*	
C22	0.5311 (4)	0.7810 (3)	0.2934 (3)	0.0336 (8)	
C23	0.5920 (4)	0.7872 (4)	0.4156 (3)	0.0378 (8)	
C24	0.6642 (7)	0.6950 (5)	0.4440 (4)	0.0690 (14)	
H24A	0.6945	0.7098	0.5267	0.104*	
H24B	0.7576	0.7121	0.4218	0.104*	
H24C	0.5861	0.6049	0.4013	0.104*	
O1	0.3522 (3)	0.5619 (2)	0.7790 (2)	0.0421 (6)	
O2	0.3737 (4)	0.7706 (3)	0.6987 (3)	0.0619 (8)	
O3	0.6876 (3)	0.8377 (3)	0.7402 (3)	0.0506 (7)	
O4	0.6767 (4)	0.6530 (3)	0.8491 (3)	0.0633 (9)	
O5	0.4601 (4)	0.9650 (3)	0.3363 (2)	0.0488 (7)	
O6	0.5830 (4)	0.8684 (3)	0.5029 (3)	0.0571 (8)	

### Atomic displacement parameters (Å^2^)

**Table:**

	*U*^11^	*U*^22^	*U*^33^	*U*^12^	*U*^13^	*U*^23^
Cu1	0.0312 (3)	0.0370 (3)	0.0406 (3)	0.0157 (2)	0.0112 (2)	0.0147 (2)
Cu2	0.0444 (4)	0.0352 (3)	0.0314 (4)	0.0241 (3)	0.0137 (3)	0.0117 (3)
C1	0.038 (2)	0.039 (2)	0.039 (2)	0.0164 (16)	0.0172 (17)	0.0062 (17)
C2	0.051 (2)	0.046 (2)	0.066 (3)	0.017 (2)	0.027 (2)	0.020 (2)
C3	0.049 (3)	0.062 (3)	0.087 (4)	0.007 (2)	0.034 (3)	0.021 (3)
C4	0.034 (2)	0.080 (4)	0.093 (4)	0.011 (2)	0.020 (2)	0.026 (3)
C5	0.038 (2)	0.078 (3)	0.064 (3)	0.023 (2)	0.012 (2)	0.021 (3)
C6	0.0350 (19)	0.048 (2)	0.039 (2)	0.0179 (17)	0.0128 (17)	0.0089 (18)
C7	0.0368 (18)	0.053 (2)	0.037 (2)	0.0237 (18)	0.0122 (16)	0.0116 (19)
C8	0.052 (2)	0.070 (3)	0.061 (3)	0.037 (2)	0.016 (2)	0.027 (3)
C9	0.036 (2)	0.043 (2)	0.043 (2)	0.0142 (17)	0.0169 (17)	0.0146 (19)
C10	0.052 (3)	0.050 (3)	0.066 (3)	0.017 (2)	0.023 (2)	0.027 (2)
C11	0.056 (3)	0.056 (3)	0.079 (4)	0.003 (2)	0.029 (3)	0.022 (3)
C12	0.035 (2)	0.073 (3)	0.084 (4)	0.003 (2)	0.016 (2)	0.020 (3)
C13	0.037 (2)	0.078 (3)	0.057 (3)	0.021 (2)	0.008 (2)	0.023 (3)
C14	0.0334 (19)	0.047 (2)	0.038 (2)	0.0156 (17)	0.0107 (16)	0.0122 (18)
C15	0.037 (2)	0.055 (2)	0.037 (2)	0.0239 (18)	0.0115 (17)	0.0163 (19)
C16	0.057 (3)	0.081 (3)	0.066 (3)	0.038 (3)	0.014 (2)	0.040 (3)
C17	0.0349 (18)	0.0349 (19)	0.035 (2)	0.0156 (16)	0.0097 (16)	0.0073 (17)
C18	0.048 (2)	0.050 (2)	0.038 (2)	0.0260 (19)	0.0090 (18)	0.0129 (19)
C19	0.052 (2)	0.049 (2)	0.031 (2)	0.0178 (19)	0.0122 (18)	0.0062 (19)
C20	0.054 (2)	0.039 (2)	0.041 (2)	0.0181 (18)	0.0202 (19)	0.0031 (18)
C21	0.043 (2)	0.034 (2)	0.052 (3)	0.0194 (17)	0.0206 (19)	0.0093 (18)
C22	0.0306 (17)	0.0305 (18)	0.039 (2)	0.0132 (14)	0.0133 (15)	0.0089 (16)
C23	0.0389 (19)	0.0342 (19)	0.046 (2)	0.0214 (16)	0.0170 (17)	0.0123 (17)
C24	0.100 (4)	0.081 (3)	0.055 (3)	0.071 (3)	0.024 (3)	0.023 (3)
O1	0.0376 (14)	0.0390 (14)	0.0541 (17)	0.0187 (11)	0.0173 (12)	0.0189 (13)
O2	0.0529 (17)	0.065 (2)	0.074 (2)	0.0300 (15)	0.0214 (16)	0.0286 (18)
O3	0.0351 (14)	0.0563 (17)	0.065 (2)	0.0183 (13)	0.0134 (13)	0.0364 (16)
O4	0.0519 (18)	0.065 (2)	0.079 (2)	0.0271 (16)	0.0203 (16)	0.0362 (18)
O5	0.079 (2)	0.0494 (16)	0.0350 (15)	0.0463 (15)	0.0201 (14)	0.0154 (13)
O6	0.0668 (19)	0.0625 (19)	0.0566 (19)	0.0401 (16)	0.0235 (16)	0.0246 (16)

### Geometric parameters (Å, °)

**Table:**

Cu1—O1	1.916 (3)	C11—C12	1.378 (7)
Cu1—O3	1.916 (3)	C11—H11	0.9300
Cu1—O2	1.934 (3)	C12—C13	1.375 (7)
Cu1—O4	1.947 (3)	C12—H12	0.9300
Cu2—O5^i^	1.906 (3)	C13—C14	1.423 (5)
Cu2—O5	1.906 (3)	C13—H13	0.9300
Cu2—O6^i^	1.952 (3)	C14—C15	1.460 (6)
Cu2—O6	1.952 (3)	C15—O4	1.311 (5)
C1—O1	1.319 (4)	C15—C16	1.518 (5)
C1—C6	1.430 (5)	C16—H16A	0.9600
C1—C2	1.432 (6)	C16—H16B	0.9600
C2—C3	1.379 (6)	C16—H16C	0.9600
C2—H2	0.9300	C17—O5	1.325 (4)
C3—C4	1.384 (7)	C17—C18	1.417 (5)
C3—H3	0.9300	C17—C22	1.429 (5)
C4—C5	1.378 (7)	C18—C19	1.380 (6)
C4—H4	0.9300	C18—H18	0.9300
C5—C6	1.431 (5)	C19—C20	1.401 (6)
C5—H5	0.9300	C19—H19	0.9300
C6—C7	1.467 (6)	C20—C21	1.380 (6)
C7—O2	1.311 (5)	C20—H20	0.9300
C7—C8	1.519 (5)	C21—C22	1.430 (5)
C8—H8A	0.9600	C21—H21	0.9300
C8—H8B	0.9600	C22—C23	1.471 (5)
C8—H8C	0.9600	C23—O6	1.313 (5)
C9—O3	1.321 (4)	C23—C24	1.520 (5)
C9—C14	1.428 (5)	C24—H24A	0.9600
C9—C10	1.436 (6)	C24—H24B	0.9600
C10—C11	1.374 (7)	C24—H24C	0.9600
C10—H10	0.9300		
			
O1—Cu1—O3	173.38 (12)	C13—C12—H12	120.3
O1—Cu1—O2	91.92 (12)	C12—C13—C14	122.6 (4)
O3—Cu1—O2	88.35 (12)	C12—C13—H13	118.7
O1—Cu1—O4	89.17 (12)	C14—C13—H13	118.7
O3—Cu1—O4	91.05 (12)	C13—C14—C9	117.6 (4)
O2—Cu1—O4	175.72 (15)	C13—C14—C15	119.4 (4)
O5^i^—Cu2—O5	180.000 (1)	C9—C14—C15	123.0 (3)
O5^i^—Cu2—O6^i^	92.29 (12)	O4—C15—C14	121.6 (3)
O5—Cu2—O6^i^	87.71 (12)	O4—C15—C16	118.1 (4)
O5^i^—Cu2—O6	87.71 (12)	C14—C15—C16	120.3 (3)
O5—Cu2—O6	92.29 (12)	C15—C16—H16A	109.5
O6^i^—Cu2—O6	180.000 (1)	C15—C16—H16B	109.5
O1—C1—C6	125.1 (3)	H16A—C16—H16B	109.5
O1—C1—C2	116.9 (4)	C15—C16—H16C	109.5
C6—C1—C2	118.0 (3)	H16A—C16—H16C	109.5
C3—C2—C1	121.9 (4)	H16B—C16—H16C	109.5
C3—C2—H2	119.1	O5—C17—C18	116.4 (3)
C1—C2—H2	119.1	O5—C17—C22	124.8 (3)
C2—C3—C4	120.5 (5)	C18—C17—C22	118.8 (3)
C2—C3—H3	119.8	C19—C18—C17	122.2 (4)
C4—C3—H3	119.8	C19—C18—H18	118.9
C3—C4—C5	119.5 (4)	C17—C18—H18	118.9
C3—C4—H4	120.3	C18—C19—C20	119.6 (4)
C5—C4—H4	120.3	C18—C19—H19	120.2
C4—C5—C6	122.7 (5)	C20—C19—H19	120.2
C4—C5—H5	118.7	C21—C20—C19	119.7 (4)
C6—C5—H5	118.7	C21—C20—H20	120.2
C1—C6—C5	117.5 (4)	C19—C20—H20	120.2
C1—C6—C7	123.1 (3)	C20—C21—C22	122.5 (4)
C5—C6—C7	119.4 (4)	C20—C21—H21	118.7
O2—C7—C6	121.4 (3)	C22—C21—H21	118.7
O2—C7—C8	118.5 (4)	C17—C22—C21	117.2 (3)
C6—C7—C8	120.1 (3)	C17—C22—C23	122.9 (3)
C7—C8—H8A	109.5	C21—C22—C23	119.9 (3)
C7—C8—H8B	109.5	O6—C23—C22	122.3 (3)
H8A—C8—H8B	109.5	O6—C23—C24	117.6 (4)
C7—C8—H8C	109.5	C22—C23—C24	120.1 (3)
H8A—C8—H8C	109.5	C23—C24—H24A	109.5
H8B—C8—H8C	109.5	C23—C24—H24B	109.5
O3—C9—C14	124.7 (3)	H24A—C24—H24B	109.5
O3—C9—C10	117.0 (4)	C23—C24—H24C	109.5
C14—C9—C10	118.3 (4)	H24A—C24—H24C	109.5
C11—C10—C9	120.8 (4)	H24B—C24—H24C	109.5
C11—C10—H10	119.6	C1—O1—Cu1	127.9 (2)
C9—C10—H10	119.6	C7—O2—Cu1	130.4 (3)
C10—C11—C12	121.3 (4)	C9—O3—Cu1	127.4 (2)
C10—C11—H11	119.3	C15—O4—Cu1	130.0 (3)
C12—C11—H11	119.3	C17—O5—Cu2	127.9 (2)
C11—C12—C13	119.4 (4)	C23—O6—Cu2	129.0 (3)
C11—C12—H12	120.3		

Symmetry codes: (i) −*x*+1, −*y*+2, −*z*+1.
